# The beneficial role of curiosity on route memory in children

**DOI:** 10.3389/fcogn.2024.1346280

**Published:** 2024-03-14

**Authors:** Yadurshana Sivashankar, Myra Fernandes, Pierre-Yves Oudeyer, Hélène Sauzéon

**Affiliations:** ^1^Department of Psychology, University of Waterloo, Waterloo, ON, Canada; ^2^Flowers Team, INRIA, University of Bordeaux, Talence, France; ^3^BPH Bordeaux Population Health, University of Bordeaux, Talence, France

**Keywords:** intrinsic motivation, curiosity, route learning, individual differences, virtual reality

## Abstract

**Introduction:**

There has been a growing interest in the role of innate curiosity on facets of human cognition, such as in spatial learning and memory. Yet, it is unclear how state level curiosity evoked by the current environment could interact differentially with trait curiosity, to impact spatial memory performance.

**Methods:**

We assessed the influence of trait and state curiosity on route memory. Forty-two 10-year-old children with low and high-trait curiosity (20 Females; 22 Males) actively explored virtual environments that elicited varying levels of uncertainty (i.e., state-curiosity).

**Results:**

As trait curiosity increased, so did memory performance in low and high uncertainty conditions, suggesting that high-curiosity children can better recruit cognitive resources within non-optimal environments. Children with high compared to low trait curiosity also reported greater feelings of presence during exploration. Importantly, in environments with medium uncertainty, children with low trait curiosity were able to perform as well as those with high curiosity.

**Discussion:**

Results show that individual differences in trait curiosity influence route learning and these interact dynamically with state-curiosity invoked within different environments.

## 1 Introduction

Since the last decade, there has been a growing interest in the role of innate curiosity on facets of human cognition, such as in spatial learning and memory (Gottlieb and Oudeyer, 2018). Early definitions of curiosity by William James (Buheji, 2019; p. 14) describe it as a “*higher, more intellectual form of impulse”* that internally drives children to explore their external environment to acquire new information. States of curiosity evoked by exploratory activities or stimuli that are surprising, novel, and of intermediate complexity, foster both spontaneous exploration and active learning in children, young as well as older adults (Oudeyer, 2007; Sakaki et al., 2018; for reviews: see Gruber et al., 2014). For example, Stahl and Feigenson (2015) observed that infants created stronger associations between sounds/words and visual objects in a context where object movements violated the expected laws of physics (i.e., a context that contradicted one's internal expectations about a target event). The primary objective of the current study was to create and use an experimental paradigm that assessed the influence of trait curiosity on spatial learning and memory, in environments that elicited varying levels of uncertainty (i.e., state curiosity is trigged by environment's properties that motivate exploratory behavior to acquire new knowledge; Loewenstein et al., 1992).

Prior studies investigating the influence of curiosity on memory are based on Loewenstein's *Information Gap Theory* (*IGT*; Loewenstein et al., 1992). *IGT* views curiosity as “arising when attention is focused on a gap in knowledge” (Loewenstein et al., 1992, p. 75). According to this theory, this gap produces a sense of knowledge deprivation that motivates one to seek out the missing information in order to eliminate the knowledge gap. For instance, Loewenstein et al. (1992) found that participants were more curious when there was an information deficit than when there was none, and significantly more curious when they had some knowledge about the target information.

In another study utilizing both behavioral and imaging methods, Kang et al. (2008) were among the first to use a Trivia question paradigm to show precisely the benefit of curiosity on memory. Here participants were presented with a series of trivia questions that served to elicit participants' interest to seek the correct answer. The researchers then examined how interest triggered by knowledge gaps facilitated memory for the answer. They found that adults exhibited enhanced long-term memory retention for verbal material for which they had expressed high relative to low knowledge gaps. They also observed greater neural activity in the striatum and inferior frontal cortex prior to the presentation of answers to questions evoking high-curiosity (due to there being a high knowledge gap). Moreover, when participants observed answers that did not match their predictions (i.e., a prediction error), an increase in activation in putamen and left inferior frontal cortex was observed. Using trivia questions, several studies consistently found that curiosity in knowing the answer was positively related to memory performance in people of various ages (Kang et al., 2008; Murayama and Kuhbandner, 2011; Gruber et al., 2014; Mullaney et al., 2014; McGillivray et al., 2015; Marvin and Shohamy, 2016; Fastrich et al., 2018). Importantly, using a Trivia question paradigm, recent results in children (10–14 years) further support the claim of a memory benefit from curiosity-driven learning (Fandakova and Gruber, 2021).

While a Trivia question paradigm has some advantages in terms of facilitating the study of curiosity-based memory, it has several limitations (Fastrich et al., 2018). First, questions are prepared in an *ad-hoc* manner with large discrepancies from one study to the next (content, set size), with potential item effects that have an influence on statistical inference in intra-individual analysis (Murayama et al., 2014, 2019). Second, trivia question paradigms do not allow interpretation of the curiosity state of participants before and after the answer is presented. Importantly, the amount and range of interest that trivia questions induce in participants is unclear, and it is difficult to compare the results across different studies. The ability to draw comparisons across different studies is particularly important in children where intra- and inter-individual variabilities are high. This underscores the need to design curiosity paradigms that are reliable and robust to intra- and inter-individual variability. Thus, our aim in the current study was to examine the influence of state curiosity on route memory performance (an index of spatial memory), in children who varied in trait curiosity (high vs. low).

### 1.1 Curiosity-driven exploration and its potential influence on spatial memory

Past studies have found that children are more inclined to explore a novel environment when some information about the surrounding is provided prior to exploration, compared to when little to no information is revealed (Jirout and Klahr, 2012; Kashdan et al., 2018; Baer and Kidd, 2022; Jach et al., 2022). In the systematic review by Jirout and Klahr (2012), they have defined curiosity as **“***the threshold of desired uncertainty in the environment which leads to* (joyous) *exploratory behavior”* (p. 125). Specifically, they assert that curiosity is most likely to occur when there is a “medium” or “optimal” level of uncertainty about the environment, resulting in an inverted-U shape relationship between curiosity and uncertainty. That is, children may feel overwhelmed to explore a surrounding of high uncertainty and may feel bored to explore one with minimal (low) uncertainty. They feel most interested in exploring environments eliciting medium levels of uncertainty (Jirout and Klahr, 2012). Their view also suggests that the peak of this inverted-U shape could differ as a function of individual differences, such as a child's trait level of curiosity (trait curiosity is dispositional curiosity related to personality). Children who are inherently inclined to resolve highly uncertain questions are considered high in trait curiosity (Jirout and Klahr, 2012).

To the best of our knowledge, no study in children has investigated the role of curiosity elicited by an uncertainty manipulation, on spatial memory. For example, following exploration of large-scale environments, wayfinding and ability to re-trace a route can be assessed as a proxy for spatial memory. It is well established that navigational tasks can be measured by different types of spatial knowledge, including landmark knowledge (visual entities), route or procedural knowledge (sequential order of landmarks encountered, as well as associations between landmarks and changes in direction), and survey knowledge (cognitive environment with Euclidean information allowing three dimensional representations; Chrastil and Warren, 2012 for review). Several studies highlight the important role of ‘decisional landmarks', such as those present at crossroads or reorientation points, in route memory (Michon and Denis, 2001; Meade et al., 2019). Such landmarks have been shown to improve memory in children as young as 6 years of age, as well as adults (Farran et al., 2012). Young children's (<8 years) ability to learn sequences of landmarks is worse than that of older children (12 years; Jansen-Osmann and Wiedenbauer, 2004). However, they are more inclined to use landmark knowledge as a form of directional guidance on a route memory task, revealing that this facet of navigation develops sometime before age 10 (Jansen-Osmann and Fuchs, 2006).

These differences and changes in spatial knowledge creation, in middle childhood, fit with age-related brain changes observed during this developmental period. Indeed, children between the ages of 7–12 experience a gradual maturation of the hippocampus and this corresponds to an increase in use of symbolic spatial representations, such as environments and models (Vasilyeva and Lourenco, 2012), suggesting that middle childhood is an important developmental period for spatial memory processing (Howard-Jones and Demetriou, 2009). Gruber and Fandakova (2021) proposed that developmental changes that occur in neural circuits of the neocortex might also enhance the benefit of curiosity on route memory. That is, in early childhood, the curiosity-reward circuit may be more directly related to the memory-hippocampal circuit. For example, Gruber et al. (2019) suggested that hippocampal-dependent memory for curiosity-related information is enhanced because it engages attention, exploration, and information-seeking behavior. This neurocognitive framework of curiosity's effect on memory stresses the interplay between curiosity and the so-called reward network in the brain, whereby curiosity enhances memory through the release of dopamine in the hippocampal memory system (Lisman and Grace, 2005; Düzel et al., 2010; Shohamy and Adcock, 2010). In light of these findings, we believe that hippocampal-mediated memory for a route traveled (Jansen-Osmann and Wiedenbauer, 2004) could be better remembered, if a curiosity-state is induced.

### 1.2 Current study

**Figure 1:**
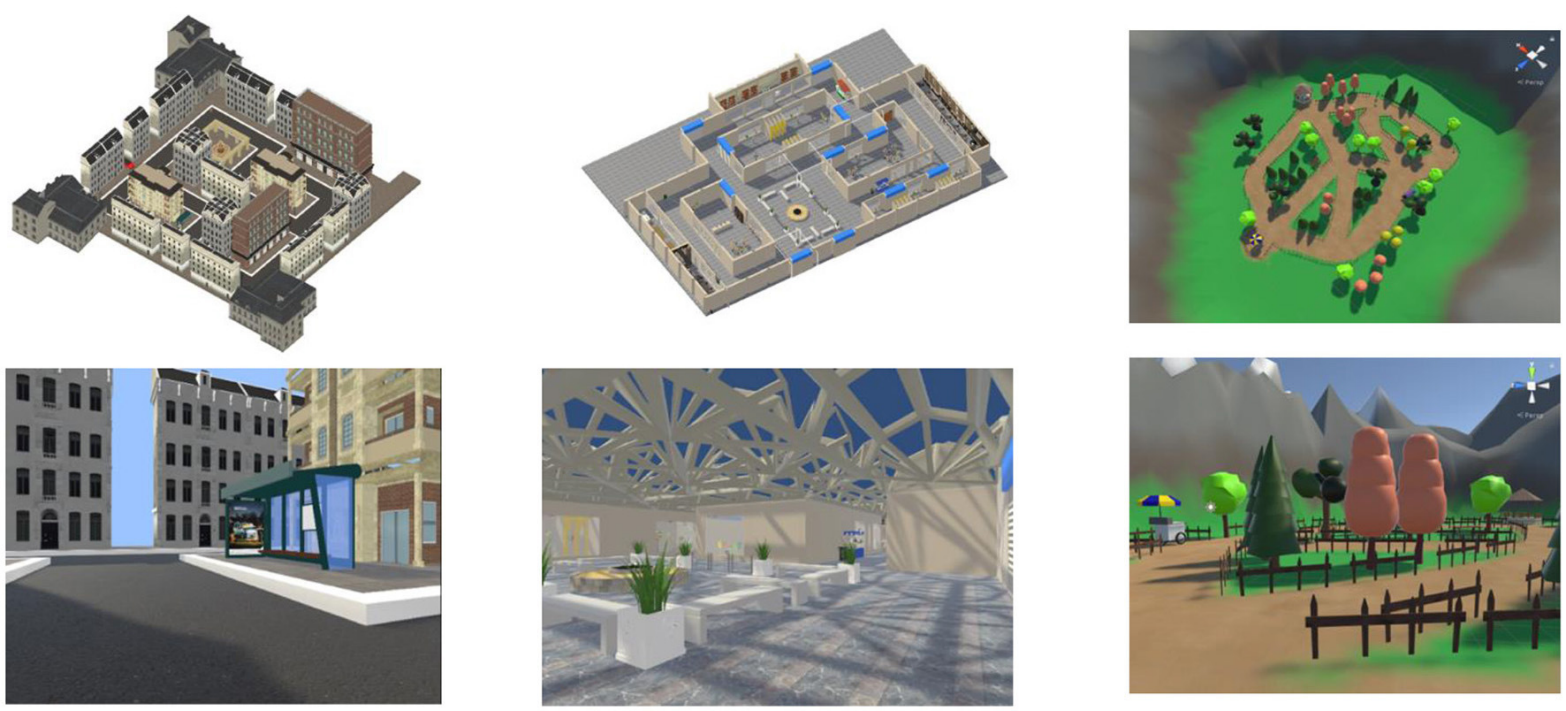
Bird's-eye view (top row) and a first person's view (bottom row) of the three styles of virtual environments. Participants only experienced the environments from a first-person perspective. **(A)** City. **(B)** Mall. **(C)** Park.

In the current study, we constructed a game-based virtual reality (VR) program consisting of nine different environments containing varying levels of uncertainty (low, medium, high). Children with different levels of trait curiosity explored these environments, after which memory for the route traveled was assessed. As reviewed by Jirout (2020), several studies have demonstrated that the presence of uncertainty leads to higher engagement (Howard-Jones and Demetriou, 2009) of environment exploration, and greater information seeking (e.g., Litman et al., 2005; Jirout and Klahr, 2012). Similar to the experimental paradigm used by Jirout and Klahr (2012), we manipulated state curiosity within each environment by increasing the level of uncertainty (i.e., low, medium, and high) in correctly predicting the appearance of a cartoon character when exploring a path. That is, at each three-way intersection (see Figure 1), participants selected a path to explore based on the cartoon character they predicted they would encounter during exploration of the chosen path. All participants experienced all three levels of uncertainty conditions (within-subject factor).

In the *low uncertainty condition*, the presence of only one image provided participants a 100% success rate in correctly predicting the appearance of a cartoon character during exploration. In the *medium uncertainty condition*, participants chose one character from three images shown prior to entering a path (~33% success rate), and in *the high uncertainty condition*, seven images were displayed (~14% success rate) at the entrance of paths making up an intersection. The process of observing and selecting characters at the entrance of paths was repeated during the entire duration of exploring an environment. Therefore, in the medium and high-uncertainty conditions, there was greater ambiguity in terms of which character would appear during exploration. Based on past findings, we predicted that greater ambiguity in predicted presence of the desired target stimuli (cartoon characters) in our medium and high uncertainty environments would evoke greater state curiosity in children, and subsequently enhance memory for spatial routes (Kreitler et al., 1975; Jirout and Klahr, 2012; Wu and Chen, 2016; Gruber et al., 2019).

Further, we also assessed individual differences in spatial navigation, digital media use, and importantly trait curiosity to determine whether these measures correlated with how well participants remembered the routes explored. Trait curiosity was measured using a trait curiosity questionnaire (*Interest and Deprivation Type Epistemic Curiosity Scale*; *IDEC*, Piotrowski et al., 2014). This questionnaire allowed us to determine whether trait curiosity altered the influence of our uncertainty manipulation (state curiosity) on spatial learning (Litman et al., 2005). To this end, we relied specifically on the scores from the Interest-type subscale of the *IDEC* questionnaire to stratify our sample into high and low trait-curiosity groups (i.e., between-subjects factor).

The Interest and Deprivations subscales are two distinct components of Epistemic Curiosity (Piotrowski et al., 2014). The Interest-type (I-type) subscale of the *IDEC* questionnaire correlates positively with openness and tolerance to ambiguous information (Litman et al., 2005). In contrast, endorsement of Deprivation-type (D-type) items (e.g., frustrated if I can't figure out a problem) signify feelings of perplexity or frustration due to having an incomplete understanding of something, or lacking the solution to a specific problem when individuals have partial knowledge about a phenomenon (e.g., “tip-of-the-tongue” responses to questions; Litman et al., 2005). Furthermore, I-type subscale of Epistemic Curiosity (EC) has previously been shown to be positively correlated with intrinsic motivation states, whereas D-type EC is related to both intrinsic and extrinsic motivation states (Litman et al., 2005). Therefore, relying on the I-type subscale allowed us to capture children who are either high or low in trait curiosity. We predicted that children who scored high on I-type items would be more likely to enjoy exploration and effectively work through problems containing ambiguous information, relative to children who scored low on this subscale (Litman et al., 2005). In addition, the properties of the *IDEC* questionnaire are well validated (Litman et al., 2005). Thus, we reasoned that our choice of using the I-type items to stratify our sample into the two groups (low vs high-trait curiosity) was theoretically sound. Further, our choice of classifying children who enjoy uncertainty and explorative behavior, as those with high trait-curiosity, is also in line with Jirout and Klahr's (2012) definition of curiosity. In addition to trait curiosity, we predicted that state curiosity manipulated by our uncertainty manipulation would interact differently with children with low and high trait curiosity to influence route memory performance. Based on prior work by Jirout and Klahr (2012), we predicted children with low-trait curiosity to benefit the most from a medium uncertainty condition, compared to both the low and high conditions (i.e., inversed-U shape curve). In contrast, we anticipated children with high curiosity to benefit from the high uncertainty condition, relative to a condition of low uncertainty.

To sum up, we constructed a game-based experiment using virtual environments, from which uncertainty of motivational objects was manipulated to influence state curiosity. We manipulated state curiosity by increasing the level of uncertainty in correctly predicting the appearance of a cartoon character, as participants explored a path in a virtual environment. We then measured the effect of state curiosity created in these environments, on spatial memory for routes traveled, in children with low and high trait curiosity.

## 2 Methods

### 2.1 Participants

Forty-two children [20 females (*M*_age_ = 10.25, *SD* = 0.55); 22 males (*M*_age_ = 10.23, *SD* = 0.53)] enrolled in fourth grade of primary school (within municipalities in and around the city of Bordeaux in the New-Aquitaine region of France), between the ages of nine and 11 were recruited (on volunteer basis) to participate in the study. Initially, there were six more children in the sample, but these children were not able to complete the study due to symptoms of simulator sickness, and failure to follow instructions.

We conducted a posteriori power analysis using the G^*^Power software according to a mixed ANOVA design with both between and repeated measures. The selected sample ensured power of 0.95 (at *p* = 0.05) to detect medium Curiosity group x Uncertainty condition interactions (*F* = 0.252) in a 2 (curiosity group) x 3 (uncertainty condition) ANOVA. The *Research Ethics Committee* approved all study procedures. Written informed consent was obtained from all participants and their parents prior to experimentation. Data collection took place between February and July of 2021.

Before the experiment began in VR, we asked the parents or guardians of participants to complete the *IDEC Scale* for young children, as a measure of trait curiosity (Piotrowski et al., 2014). Based on the scores obtained on the *Interest subscale* of the *IDEC* scale, we divided participants into two groups: 'high curiosity' and 'low curiosity' (High Curiosity group included children scoring above the median value of 15; Low Curiosity group included children scoring below this median value), see Table 1. Individual difference measures were also collected to probe for personal interest in exploring new environments, visual-spatial functioning, digital usages, and simulator sickness (see Table 1). These measures were included to control for their possible influence on route memory score within our VR paradigm.

**Table 1:** Mean descriptive characteristics (standard deviation in parentheses) of children with low and high trait-level curiosity.

**Characteristic**	**Low curiosity group *n* = 21**	**High curiosity group *n* = 21**
Age	10.33 (0.58)	10.19 (0.51)
Sex	15 Boys/6 Girls	8 Boys/ 13 Girls
Interest type-curiosity trait	13.38 (0.33)	17.42^***^ (0.52)
Deprivation type-curiosity trait	13.66 (2.87)	15.23 (2.49)
Total curiosity trait	27.04 (3.55)	32.66^***^ (3.27)
*SBSODS*	4.71 (0.85)	4.80 (0.83)
*HVO*	22.76 (2.55)	20.90 (3.78)
*Corsi BTT Forward*	4.90 (0.87)	4.95 (0.80)
*Corsi BTT Backward*	4.78 (0.90)	4.78 (1.05)
Digital Experience	9.69 (4.23)	10.33 (2.74)
VR Interest	8.73 (0.23)	8.36 (0.86)
Character appeal	9.64 (0.89)	9.40 (1.56)
*Child SSQ*	2.71 (3.24)	3.00 (1.77)

SBSODS, Santa Barbara Sense-of-Direction Scale; HVO, Hooper Visual Organization; Corsi BTT (F) = Forward Corsi Block Tapping Task, Corsi BTT (B) = Backward Corsi Block Tapping Task, Child SSQ, Child Simulator Sickness Questionnaire; Asterisks denote significant group differences, ^***^ (p <0.001.

Visuospatial functioning was assessed before exposure to our VR experiment using three test measures. It was critical that we assessed participants ability in visuospatial tasks to later determine whether inherent visuospatial skills influenced route memory performance, beyond our experimental manipulation. The spatial visualization ability of participants was evaluated using the *Hooper Visual Orientation* (*HVO*) test (Boyd, 1981). This test contains 30 lines drawings of common objects that are portrayed as having been cut up and misaligned; participants are instructed to mentally rotate and piece together the visual information, to identify the depicted object. Visuo-spatial short term and working memory were assessed using the *Corsi Block-Tapping Task* (Arce and McMullen, 2021) to establish the forward and backward visuospatial span. Finally, spatial orientation ability of participants was evaluated with the *Santa Barbara Sense-of-Direction* (*SBSDS*) scale (Hegarty and Waller, 2006). Each participant rated their endorsement of 15 statements about spatial orientation in everyday life, such as “I am good at giving directions,” indicating whether they strongly agreed or disagreed, using a 7-point Likert-scale. After reverse scoring the items, the responses were summed and divided by 15 to derive an average score between 1 to 7.

Participants then completed a *Digital Experience* (Moffett et al., 2021) questionnaire composed of three items. These items included the following questions: “How often do you use electronic devices (smartphones, tablets, consoles, and computers)?”, “How often do you play video games?”, and “How often do you play computer games that involve VE technology (e.g., flight or driving simulators)?” Participants rated each item from 0 to 7, to obtain a maximum score of 21 on this questionnaire.

We also assessed participants' *interest* in exploring virtual environments using a single item questionnaire completed prior to VR immersion (i.e., “On a scale from 1[lowest] to 10 [highest], how much do you enjoy discovering new environments?). Similarly, *character appeal* was assessed using a single item questionnaire completed after exposure to VR immersion [i.e., Did you like the characters? using a scale ranging from Not at all (0) to Extremely (10)].

The *Child Simulator Sickness Questionnaire* (*SSQ*; Hoeft et al., 2003) was used to assess the severity of VR sickness experienced during the VR experiment. The *SSQ* measured oculomotor discomfort, disorientation, nausea, and fatigue experienced as a result of VR exposure. We scored this questionnaire by assigning each question a value based on the response. Each “No” response received a score of 0, each “A little” response received a score of 1, and each “A lot” response received a score of 2. A score of 1 or 2 reported for an item on the *SSQ* questionnaire indicated the presence of simulator sickness for the child.

Overall, the two children's groups significantly differed only on the curiosity I-type trait score (global curiosity-trait score), but not on other variables (age, sex, spatial functioning, digital experience, character appeal, and *SSQ*). Importantly, the two groups did not differ in terms of their prior interest toward immersive VR.

### 2.2 Materials

A computer-run VR application was designed on the 3D Unity engine (ver. 2017.2.0.f3; see specifications on https://www.vive.com/ca/). This application allowed active exploration of virtual environments with a lightweight HTC VIVE mobile headset and handheld controller, with continuous button press and heading rotation, for an easy-to-use control interface. The VR application included nine Virtual Environments (VEs) previously used for assessing route memory in adults (Meade et al., 2019). The nine VEs consisted of three types of styles: city streets, mall corridors, and park trails (see Figure 1). They were all topographically similar with an average size of 234 m^2^, with six intersections in each of the nine environments.

**Figure 2:**
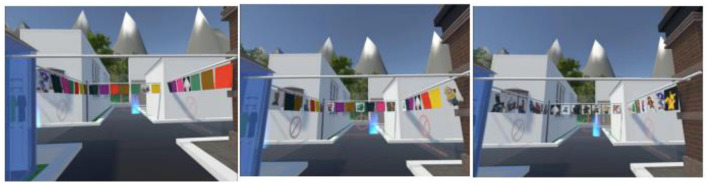
From left to right: low uncertainty (one character, 100% certainty of an encounter), medium uncertainty (three possible characters, 33% certainty of an encounter), and high uncertainty (seven possible characters, 14% certainty of an encounter).

The VR application included three exploration conditions (study phases) that varied in terms of uncertainty level (low, medium, and high) at intersection points (see Figure 2). At each intersection, we displayed pictures of cartoon characters, or solid colors, on seven different flags aligned horizontally at a height of 3 meters. We randomly assigned the location of a carton image on one of the seven flags at each intersection, in order to encourage the participant to perform a visual search. In the *Low Uncertainty Condition*, only one character was displayed on one of the flags at the entrance to each intersection; the other six flags were simply a variety of different colors. In the *Medium Uncertainty Condition*, we showed three cartoon images at the entrance to each intersection (with the remaining flags varying in color). In the *High Uncertainty Condition*, we showed seven cartoon character images (Superman, Spiderman, Batman, Spider-Gwen, Hulk, Ironman and Black Panther) at the entrance to each intersection. Here participants could encounter any one of the seven cartoon characters on the road as they explored the selected path.

### 2.3 Measures

#### 2.3.1 VR-based measures for each uncertainty condition

**Figure 3:**
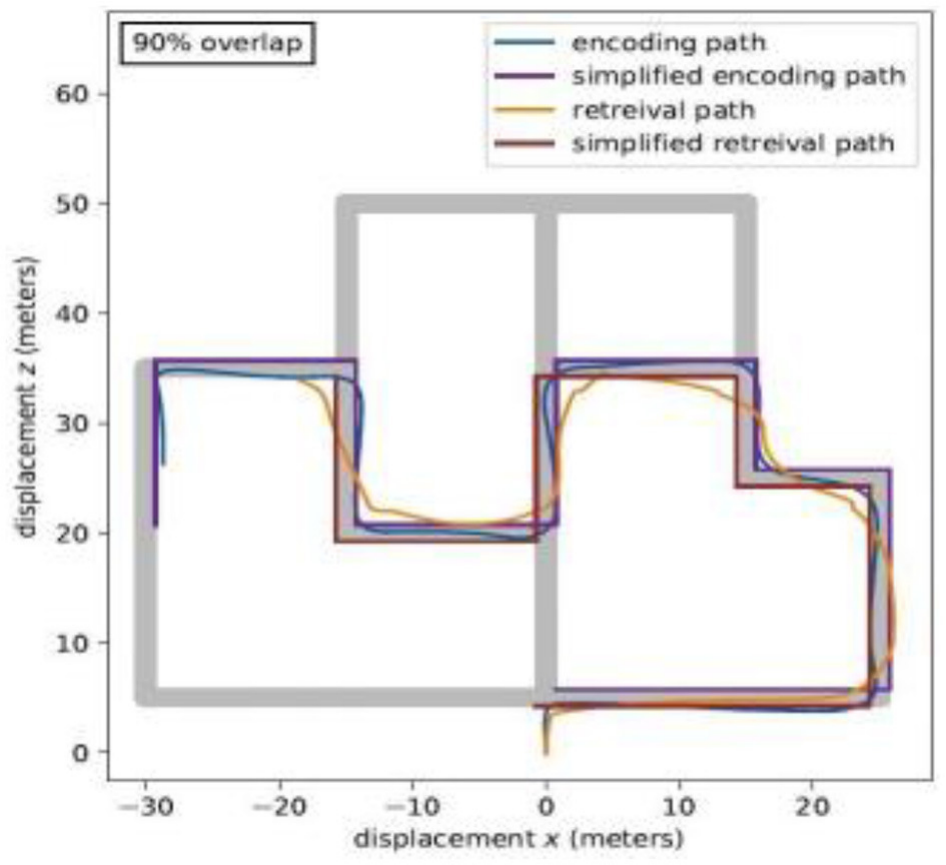
Example of the path traveled in one of the virtual environments at encoding and again at retrieval.

The aim of the study was to assess the influence of the uncertainty manipulation (low, medium, and high; within-subject factor) on spatial route memory performance in both High and Low Trait-Curiosity children (between-subjects factor). The primary dependent variable of interest was the accuracy in re-tracing the same route at retrieval that had been traveled during encoding (see Figure 3). To obtain this measure of spatial memory performance, the overlap between the routes traveled by each participant at encoding and retrieval, for each condition, was calculated to produce a “*percent overlap*” value for each environment (detailed procedure explained in Meade et al., 2019). A higher “percent overlap” value is indicative of better memory for the routes traveled at encoding. The *route overlap score* was then corrected for the number of direction changes for each participant. The number of direction changes was counted for each study phase of each participant. The following formula was applied: corrected Wayfinding score = (Wayfinding performance) ^*^ (Number of direction changes/6). Six is the maximum number of changes of direction that can be made by a participant.

Participants were also invited to rate their *intrinsic motivation* elicited by each uncertainty conditions using two questions with a rating scale from 0 to 6 [e.g., (1) Was the low uncertainty condition your preference? Participants self-rated on a scale from 0 (not at all) to 4 (absolutely); (2) Was the low uncertainty condition the most satisfactory to make your predictions of character appearances at intersections? Participants self-rated on a scale from 0 (not at all) to 4 (absolutely)]. Each participant's *intrinsic motivation* score was tabulated as the sum of ratings from these two questions, for an overall score ranging from 0 to 8. We assessed intrinsic motivation to later ensure that our experimental manipulation of uncertainty worked as intended. That is, we predicted both the high and medium uncertainty conditions to have elicited greater motivation for exploration, compared to the low uncertainty condition.

#### 2.3.2 Measures administered post-VR immersion

We administered the *Igroup Presence Questionnaire (IPQ)* to determine the extent to which participants felt present while exploring the VR environments (Darken et al., 1999; Schubert et al., 2001). Participants were instructed to rate each statement (e.g., “In the computer generated world, I had a sense of being there”, I felt present in the virtual space”) from a scale from 1 (not at all) to 7 (very much).

Finally, participants completed a two-item questionnaire evaluating the *emotional experience* elicited by the characters displayed during environment exploration in VR; With (1) *Perceived positive emotion for succeeded predictions* [i.e., Were you happy when the character you chose appeared?] rated on a scale ranging from −3 (extremely negative) to 3 (extremely positive) and (2) *Perceived negative emotion for failed predictions* [i.e., Were you disappointed when the character you chose did not appear?] rated on a scale ranging from −3 (extremely negative) to 3 (extremely positive). Again, the scores from the two items were averaged, thereby providing an ‘emotional valence score' ranging from −3 (extremely negative) to 3 (extremely positive). We included these questionnaires to assess whether the subjective VR experience across the three different uncertainty conditions influenced memory for the paths traveled.

### 2.4 Procedure

Throughout the entire VR experiment, participants were seated in a comfortable chair that could turn a full 360° circle, which was placed in the center of a testing room. Following verbal instruction of how to use the VR headset, participants were given unlimited time to complete a training phase with the VR equipment, designed to help them learn how to navigate in the virtual space. A single handheld VR controller was sufficient to navigate in the environment. To move forward, participants were instructed to press the Trackpad Button of the controller. To change their direction, participants had to rotate their head in the desired direction, as well as physically rotate in the spinning chair with their legs. We advised participants to take as much time as needed in the training phase in order to become comfortable with the environment. Participants practiced aiming at flags with the hand controller and selected flags with the Trigger of the controller. The flag selection action allowed us to track within our VR experiment, which character was chosen and predicted by the child as the one who will appear during the new direction, and ensured that the VR application reflected our manipulated probabilities [100% (low-uncertainty), 33% (medium-uncertainty), and 14% (high-uncertainty)] for predictions. Immediately following the training phase, the experimental session began.

There were two main phases during the VR experiment: A study/encoding phase (90 secs) followed by a filler task (60 secs), and then a retrieval phase (90 secs) in which participants re-traced the exact route taken at encoding. During the study phase (i.e., the route of the path taken at encoding), participants actively explored nine environments for 90 s each (i.e., they decided the path of travel and exerted full motor control through the track pad of the VR controller). After exploration, a message indicated the end of the exploration/study phase and participants were then asked to count backwards as a filler; this was done to prevent rehearsal of the route just traveled. Immediately after counting backwards, participants were placed in the same VR environment again, at the same starting point as in the Study phase, and were instructed to re-trace their exact path traveled, within the 90 s allotted for each retrieval trial.

Each participant explored a total of nine environments, with the retrieval test for each one immediately following its encoding phase. Encoding trials were blocked by condition type (three environments in the Low Uncertainty, three environments in the Medium Uncertainty, and three environments in the High Uncertainty conditions). The order of the three uncertainty conditions was counterbalanced across participants. The total time spent in VR was 36 min. Two children were evaluated during a given experimental session. While the first participant was immersed in VR, the other completed the neuropsychological tests as well as the questionnaires. Then the roles reversed.

## 3 Results

### 3.1 Intrinsic motivation score and route memory performance

#### 3.1.1 Intrinsic motivation ratings

**Figure 4:**
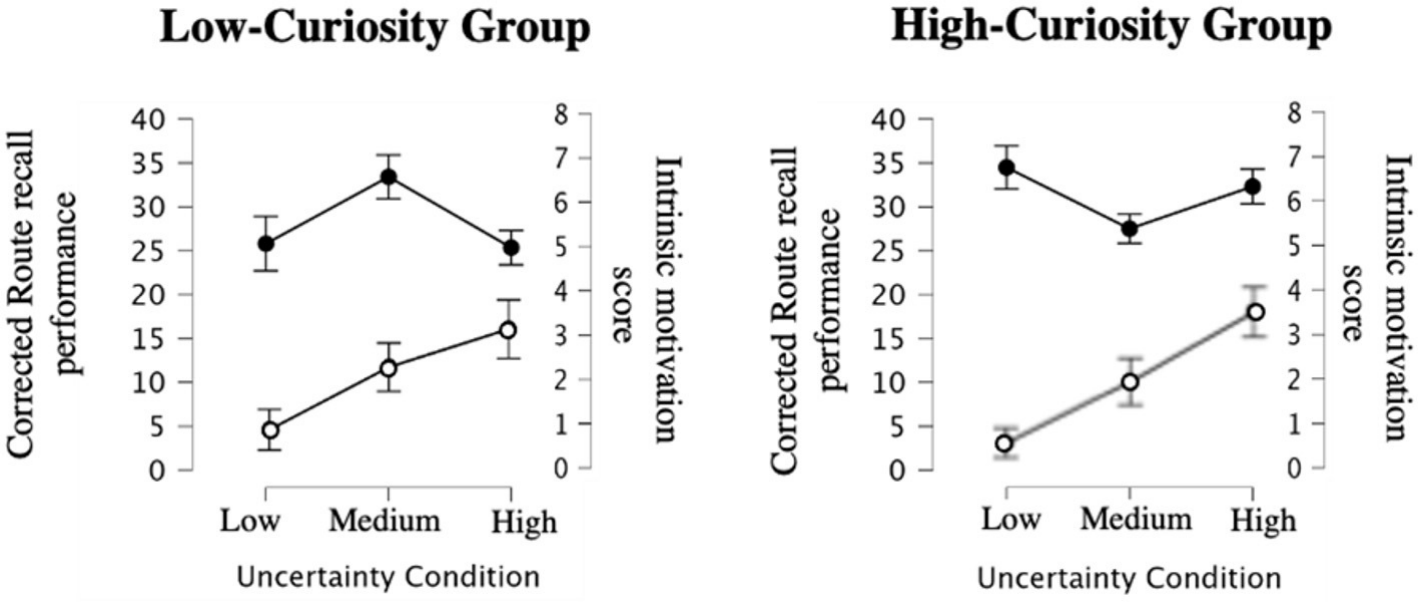
Route recall score (black circles) and intrinsic motivation score (white circles) in low-and high-curiosity groups as a function of the three uncertainty conditions (low, medium and high).

We conducted analyses to serve as a manipulation check that our uncertainty conditions elicited varying levels of motivation to explore spatial routes. We conducted a 2 (Curiosity Group: low, high; between-subjects) × 3 (Uncertainty Condition: low, medium, high; within-subject) mixed ANOVA examining participants' self-reported motivation “to explore and correctly predict character appearances at intersections” as the dependent variable (see Table 2 for means; see Figure 4). We found a significant main effect of Uncertainty Condition [*F*_(2, 80)_ = 14.36, *MSE* = 131.52, *p* < 0.001, $\eta_{p}^{2}$ = 0.26]. Participants rated both the high [*M* = 4.62, *SE* = 0.39; *t*_(80)_ = −5.33, *SE* = 0.66, *p* < 0.001] and medium uncertainty [*M* = 3.14, *SE* = 0.65; *t*_(80)_ = −3.09, *SE* = 0.66, *p* = 0.005] conditions to have elicited greater motivation compared to the low uncertainty condition (*M* = 1.09, *SE* = 0.39). Further, motivation scores were the highest in the high, relative to the medium uncertainty condition [*t*_(80)_ = −2.23, *SE* = 0.66, *p* = 0.031] (see Table 2 for Means).

**Table 2:** Mean motivation scores and VR experience ratings (standard deviation in parentheses) in children with low and high trait curiosity within each uncertainty condition.

	**Low curiosity group**	**High curiosity group**
**Intrinsic motivation scores (*****max. score** =* **8)**		
Low uncertainty condition	0.86 (1.52)	0.42 (0.74)
Medium uncertainty condition	2.19 (2.04)	1.80 (2.15)
High uncertainty condition	3.00 (2.51)	3.81 (2.42)
**Self-rated VR experience**		
*IPQ* total score	7.90 (8.33)	17.00^*^ (07.21)
Character prediction-related Emotional valence score	2.00 (0.84)	1.85 (1.10)

IPQ, Igroup Presence Questionnaire; ^*^ significant group difference (p <0.05).

There were no significant main effect of Curiosity Group [*F*_(1, 40)_ = 0.45, *MSE* = 0.51, *p* > 0.50], or Curiosity Group X Uncertainty Condition Interaction [*F*_(2, 80)_ = 0.54, *MSE* = 4.98, *p* > 0.50]. These results indicate that our manipulation of uncertainty did indeed elicit varying levels of motivation, and these did not differ across the Curiosity groups.

#### 3.1.2 Route overlap (memory) performance

We then conducted a 2 (Curiosity Group: low, high; between-subjects) × 3 (Uncertainty Condition: low, medium, high; within-subject) mixed ANOVA examining Corrected Route Overlap score as the dependent variable. See Table 3 for means.

**Table 3:** Mean route memory scores (standard deviation in parentheses) in children with low and high trait curiosity within each uncertainty condition.

	**Low curiosity group**	**High curiosity group**
**Route recall score (** ***percent overlap*** **)**		
Low uncertainty condition	25.80^*^(12.51)	34.49 (10.68)
Medium uncertainty condition	33.42 (14.32)	27.51 (7.65)
High uncertainty condition	25.35^*^ (9.16)	32.32 (10.29)

^*^significant group difference (p <0.05).

There was no significant main effect of Uncertainty Condition on Route Memory score [*F*_(2, 80)_ = 0.28, *MSE* = 113.61, *p* = 0.761, $\eta_{p}^{2}$ = 0.01], nor was there a significant effect of Curiosity Group [*F*_(1, 40)_ = 2.46, *MSE* = 135.01, *p* = 0.125, $\eta_{p}^{2}$ = 0.06]. There was, however, a significant Group X Uncertainty Condition interaction [*F*_(2, 80)_ = 5.88, *MSE* = 113.61, *p* = 0.004, $\eta_{p}^{2}$ = 0.13] (see Figure 4).

To better understand the interaction, we conducted a paired sample *t*-test comparing Route Overlap Memory score in each of the three Uncertainty conditions (low, medium, and high), separately for each Group (Low and High-Trait-Curiosity). As predicted, for the Low Curiosity Group, participants' memory for routes traveled tended to be the best in the medium uncertainty condition (*M* = 33.42, *SD* = 14.32) relative to low (*M* = 25.80, *SD* = 12.51), *t*_(20)_ = 1.77, *SE* = 4.32, *p* = 0.044, Cohen's *d* = −0.38, and high conditions (*M* = 25.35, *SD* = 9.16), *t*_(20)_ = 3.04, *SE* = 2.65, *p* = 0.006, Cohen's *d* = 0.67. Performance in the low and high conditions did not differ significantly *t*_(20)_ = 0.12, *p* > 0.900.

In contrast, for the High Curiosity Group, participants performed worst in the medium uncertainty condition (*M* = 27.51, *SD* = 7.65) relative to both low (*M* = 34.49, *SD* = 10.68), and high (*M* = 32.32, *SD* = 10.29) conditions, *t*_(20)_ = 2.31, *SE* = 3.02, *p* = 0.031, Cohen's *d* = 0.51, and *t*_(20)_ = 2.18, *SE* = 2.21, *p* = 0.041, Cohen's *d* = −0.48, respectively. We did not find any significant differences between low and high uncertainty conditions *t*_(20)_ = 0.64, *p* > 0.500.

Looking at the interaction in another way, independent samples *t*-tests revealed that Low Trait-Curiosity participants performed worse than High Trait-Curiosity participants in both the low *t*_(41)_ = −2.42, *SE* = 3.59, *p* = 0.020, Cohen's *d* = −0.75, and high uncertainty conditions, *t*_(41)_ = −2.32, *SE* = 3.01, *p* = 0.026, Cohen's *d* = −0.72. However, the two groups did not significantly differ in the medium uncertainty condition (*p* > 0.10).

**Figure 5:**
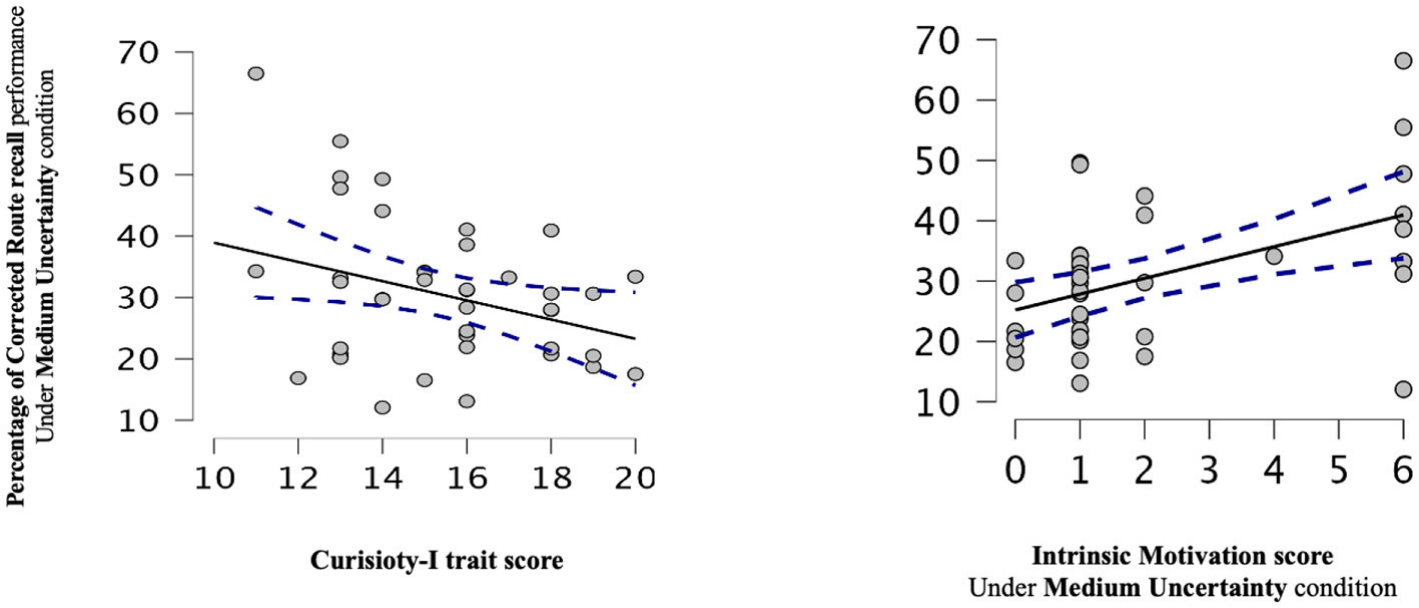
Scatter plots illustrating the relation between the corrected route recall under medium uncertainty condition and curiosity-I trait score **(left panel)** and the intrinsic motivation score elicited for this condition **(right panel)**.

Given our goal of determining whether route memory performance, intrinsic motivation, and sense of presence in VR (measured by *IPQ*) were related to various individual difference indices pertaining to spatial navigation and digital media use, we ran a series of three Pearson correlations with α = 0.05 and with adjusted α = 0.02 (Bonferroni adjustment for 3 correlations was applied when determining significance), examining *memory performance* and *intrinsic motivation* in each of the three uncertainty conditions, we observed a positive significant relation only for the medium uncertainty condition(*r* = 0.47, *p* = 0.002 (see Figure 5). In addition, a negative relation was observed between the curiosity-I trait score and route memory performance for the medium curiosity condition (*r* = −0.32, *p* = 0.033, with α = 0.03), providing additional evidence to our finding that children of Low-curiosity performed best under this state. The inverse was true for those in the High-curiosity group. All other correlations were non-significant (all *p* > 0.05).

### 3.2 Group difference on measures administered post-VR immersion

The two groups differed significantly only on the *IPQ* (*Igroup Presence Questionnaire*). We found that those in the High-Trait Curiosity group reported a greater sense of presence (*M* = 17.00, *SD* = 7.21) while being immersed in VR, relative to children with Low-Trait Curiosity (*M* = 7.90, *SD* = 8.33), *t*_(40)_ = −2.16, *p* = 0.037, Cohen's *d* = −0.67 (see Table 2). In contrast, the groups did not differ in terms of their rating of emotional experience related to their correct predictions of a character's appearance *t*_(40)_ = −0.47, *p* > 0.600.

## 4 Discussion

In the last decade, there has been a growing interest in how curiosity might influence memory and learning (Gottlieb and Oudeyer, 2018). In the current study, we constructed a game-based experiment using virtual environments, from which uncertainty of motivational objects was manipulated to influence state curiosity. We manipulated state curiosity by increasing the level of uncertainty in correctly predicting the appearance of a cartoon character, as participants explored a path in a virtual environment. We measured the effect of state curiosity created in these environments, on spatial memory for routes traveled, in children with low and high trait curiosity.

Children with low and high trait curiosity were asked to actively explore virtual environments containing varying levels of uncertainty. We then assessed memory for routes traveled. Children in the high-trait curiosity group performed the best in both high and low uncertainty environments, suggesting that these children are able to benefit from non-optimal states of exploration. Importantly, in environments with medium uncertainty, children with low-trait curiosity were able to perform as well as those with high curiosity. Results highlight an important influence of individual differences on cognitive task performance (route memory) in environments with varying levels of uncertainty.

As suggested by past work, we predicted that an optimal level of uncertainty regarding the presence of cartoon characters in the medium uncertainty condition would confer the greatest benefit to route overlap, relative to low and high conditions (Kreitler et al., 1975; Jirout and Klahr, 2012; Wu and Chen, 2016; Gruber et al., 2019). These predictions were in line with the findings by Jirout and Klahr (2012), who claim that children are more inclined to explore a novel environment when some information about the surrounding is provided prior to exploration, compared to when little to no prior information is revealed. That is, a ‘medium' or optimal level of uncertainty about the environment is the most useful in eliciting levels of curiosity. Although we did not observe a significant main effect of uncertainty condition, or group differences in route memory performance, we did find a significant crossover interaction. As predicted, we found the medium uncertainty condition to benefit memory for routes, more so than the conditions (environments) with low and high curiosity – however this was true only in children of low trait-curiosity. To our surprise, children of high trait-curiosity performed the worst in the medium uncertainty condition, and higher in the low and high uncertainty conditions. Finally, we also found that those in the high-trait curiosity group reported a greater sense of presence while being immersed in VR, relative to children with low-trait curiosity.

### 4.1 Optimal uncertainty as a booster for spatial memory in children

Our pattern of findings is in line with previous findings revealing that children, youth, and adults with high relative to low trait curiosity show high academic achievement, and are often high performers on various cognitive tasks, particularly when the cognitive tasks tap on explorative processes (Von Stumm, 2013; Von Stumm and Ackerman, 2013; for review, Mussel, 2022).

Prior research in spatial learning in young adults (for reviews: Chrastil and Warren, 2012; Smith, 2019), suggests that active exploration (relative to passive where one is simply guided to a path; no volitional control of movement) enhances memory for routes traveled and location of landmarks by a way of requiring greater attention and decision-making strategies (Sauzéon et al., 2015). Research in children also shows that they must first learn how to direct attention to spatial cues when navigating, suggesting that attention is a general ability that is important in navigational tasks (Heth et al., 1997). Cornell et al. (1989) showed that encouraging 6–12-year-old children to direct attention to proximal landmarks (i.e., landmarks near the route) rather than distal landmarks (i.e., landmarks seen from far away), helped them to retrace a route more successfully. In light of the findings of the current study, we propose that in addition to attention and decision-making, curiosity-driven spatial learning is another critical component underlying the benefit of active exploration. Behavioral evidence in favor of this position is also provided by studies examining the gaming effect in children. For instance, active exploration of an environment is reported to be highly beneficial to children, if elements of gamification such as finding motivational objects are introduced into the task while exploring the environment (Fornasari et al., 2013; Proulx et al., 2016). A recent study on the PokemonGo^®^ game also revealed that greater curiosity elicited by the game increased the quality of the spatial representation of the environments explored by the children (Blasko et al., 2018).

Furthermore, neural evidence in favor of curiosity-driven spatial memory stems from the Prediction Appraisal Curiosity and Exploration (PACE) framework (Gruber et al., 2019). This neurocognitive theory suggests that curiosity can be triggered by a surprising contextual change or detection of a new context, signaled by the anterior hippocampus, or by the detection of an abstract knowledge gap, signaled by the anterior cingulate cortex (Gruber and Fandakova, 2021). Appraisal of the triggered event as negative leads to behavioral inhibition and anxiety. While, appraisal of the event as salient and valuable for the future leads to the subjective experience of curiosity and recruitment of the dopaminergic circuit, which serves to promote explorative and information seeking behavior. Thus, curiosity-induced dopaminergic neuromodulation of the hippocampus is believed to aid in the consolidation of memories for both the target stimulus and any incidental information encountered during a curiosity state. When children of low-trait curiosity made the prediction as to which character will appear as they explored the selected path, the anticipation of the cartoon character, particularly in the medium uncertainty condition, served as valuable information for future, ultimately enhancing their memory. The relatively poorer performance of this group, in the low and high uncertainty conditions, could be due to appraisal of the event as lacking a critical knowledge gap (in the low uncertainty condition) and as an overwhelming event (in the high uncertainty), leading to a reduction in engagement. These results are consistent with the findings from Jirout and Klahr (2012) who also observed that an optimal level of uncertainty peaks curiosity in children, while too little or too much information attenuates levels of curiosity as recently stressed in developmental curiosity-driven learning models (respectively, Gottlieb and Oudeyer, 2018; Gruber and Fandakova, 2021; see also Baer and Kidd, 2022).

Having a direct manipulation of curiosity on memory offers a real methodological and statistical advantage such as overcoming the limitations of pre and post subjective evaluation of curiosity states that are frequently assessed in Trivia question paradigms (Murayama et al., 2014, 2019; Fastrich et al., 2018). Additionally, the use of direct manipulation rather than subjective reports avoids the possible confound of results related to metacognitive inaccuracies, documented in children (e.g., Kuhn, 2000).

### 4.2 Curiosity, individual differences in spatial memory, and sense of presence

To the best of our knowledge, our observation of individual differences in terms of feelings of presence in a VR environment is new. Interpersonal variability in reported VR presence has been shown across sex, and video game experience (see Li et al., 2020 for review). Often these individual differences have been invoked as explanations for differences in spatial abilities. These spatial abilities cannot account for our data as no differences were observed across our curiosity groups, on small- (spatial working memory and spatial visualization) or large-scale (everyday sense-of-orientation) spatial tasks.

Alternatively, the sense-of-presence advantage in high curiosity group could reflect their increased attention-based exploratory capabilities, as suggested by past work (e.g., Jirout and Klahr, 2012; Kashdan et al., 2018; Baer and Kidd, 2022; Jach et al., 2022). As such, the present individual variability in route memory and VR presence deserves further exploration in future work in order to elucidate the specific curiosity-trait related cognitive mechanisms underlying sense of presence.

Further, as suggested by Jirout and Klahr (2012), the benefit conferred by an optimal state of curiosity on memory hinges on individual differences, that is, the trait-curiosity of the child. A critical finding of our paper is that memory in children of high-trait curiosity was worst in the medium uncertainty condition, and better in the low and high conditions. To our knowledge, this is the first study to report such a nuanced finding regarding the influence of trait curiosity on route memory. Based on the findings of our study, we can conclude that for children of high-trait curiosity, being immersed in an environment of medium uncertainty does little to benefit spatial route learning.

We reason that our results observed in children of high-curiosity are a product of the type of exploration engaged by the child, more than the curiosity state evoked by the environment (Kashdan et al., 2018; Jach et al., 2022). For example, Hsiao et al. (2017) found that high learners exhibited a higher roaming entropy, indicative of complex paths and extensive active exploration of virtual environments. In addition, children of high-trait curiosity in our sample reported a greater sense of presence while being immersed in VR, relative to children with low trait-curiosity. Presence is typically seen as a multidimensional construct, including several components such as agency, enjoyment, emotional engagement, and vividness of the virtual environment, all contributing to the overall sense of feeling present in immersive VR (Lessiter et al., 2001). As highlighted by Smith (2019) in his review, presence is a function of how much visual attention is oriented toward the virtual environment, thus offering evidence to our view that attention based explorative behavior underpins route learning in high trait-curiosity children. Future studies should precisely examine the types of explorative strategies engaged by children of high-trait curiosity in various environments (e.g., route exploration driven by a saliency of landmark) to specify the roaming strategy that confers the greatest benefit to route learning.

### 4.3 Limitations

Several limitations to our work must be highlighted and constitute new research perspectives. First, the sample size could be increased to enhance the statistical power of the results, and to allow inclusion of several additional levels of trait-curiosity. This could also allow one to conduct other statistical analyses such as structural equation modeling with which some of our assumptions could be assessed more directly (e.g., the causal links between trait curiosity, extent of spatial exploration, intrinsic motivation, sense-of-presence, and subsequent spatial memory performance). Further, in the current research, we created groups of high and low trait curiosity based on the Interest-type (I-type) subscale of the *IDEC* questionnaire. Although this method of group stratification allowed us to detect an interactive effect between trait curiosity and spatial memory performance, we recognize that this approach limits analyses of our results on a continuum.

Second, as mentioned above, the application of the statistical method of “roaming entropy” (Freund et al., 2013) for detecting differences in movement trajectories in active exploration of space could be a relevant way to investigate the effects of interpersonal variables (i.e., curiosity-trait) and of uncertainty variables (i.e., elicited curiosity) during the critical exploration phase. Nevertheless, the use of this method will require some adaptations, especially to account for differences in content and size of virtual environments.

Finally, we had not predicted individual differences related to trait curiosity on reported feelings of presence within VR. These could be better captured and understood using the *Immersive Tendencies Questionnaire* (Witmer and Singer, 1998), although it would require adaption to allow implementation in children.

### 4.4 Conclusion

Results suggest that individual differences influence cognitive task performance in environments with varying levels of uncertainty. Our primary results showed that an optimal uncertainty level exists to improve children's memory for routes traveled, and that this differs depending on a child's trait-level curiosity. For those with low trait curiosity, memory is best in medium relative to low or high uncertainty environments, whereas the inverse is true in children with high levels of trait curiosity. Of note, we showed this in children, using an immersive virtual reality paradigm, demonstrating its utility in scientific research. Second, our work highlights critical inter-individual differences pertaining to trait curiosity and spatial memory performance, which vary as a function of the level of uncertainty provided by the environment. Our findings highlight the importance of individualizing curiosity-based interventions, particularly for psychoeducational or cognitive improvement purposes. Further, an equal ratio of male and female children in our sample lends generalizability of our findings to the general population. This first study specifying the intricate role of individual differences on state curiosity and route memory suggests that future research should consider the dynamic nature of trait-curiosity on spatial performance.

## Data availability statement

The raw data supporting the conclusions of this article will be made available by the authors, without undue reservation.

## Ethics statement

The studies involving humans were approved by the National Institute for Research in Digital Science and Technology. The studies were conducted in accordance with the local legislation and institutional requirements. Written informed consent for participation in this study was provided by the participants' legal guardians/next of kin. Written informed consent was obtained from the individual(s), and minor(s)' legal guardian/next of kin, for the publication of any potentially identifiable images or data included in this article.

## Author contributions

YS: Writing – review & editing, Writing – original draft, Project administration, Methodology, Investigation, Formal analysis, Data curation, Conceptualization. MF: Writing – review & editing, Writing – original draft, Supervision, Funding acquisition. P-YO: Writing – review & editing, Writing – original draft. HS: Writing – review & editing, Writing – original draft, Supervision, Project administration, Methodology, Funding acquisition, Formal analysis, Data curation, Conceptualization.
